# Do Nursing Home Culture Change Practices Improve Residents’ Quality of Life and Promote a Person-Centered Climate?

**DOI:** 10.1093/geroni/igaa057.1228

**Published:** 2020-12-16

**Authors:** Minhong Lee, Kyeongmo Kim, Sok An

**Affiliations:** 1 Dong-eui University, Busan, Pusan-jikhalsi, Republic of Korea; 2 Virginia Commonwealth University, Richmond, Virginia, United States; 3 Korea Rural Economic Institute, Austin, Texas, United States

## Abstract

Background and Purpose: Although nursing home culture change models (e.g., Green House, The Pioneer Network) in the U.S. have been proven to improve the quality of life of the residents by providing person-centered care and home-like environment, limited efforts have been made to implement a model in Korea. The purpose of this study was to examine whether the adapted culture change model would improve the quality of life of the residents and strengthen the person-centered climate. Methods: Employing a quasi-experimental design, the PI developed and implemented a 12-session culture change enhancement program to 32 staff in four nursing homes. Data were collected from 109 nursing home residents before and after the intervention, and key measures included the quality of life scale and the person-centered climate. The paired t-test analyses were performed with SPSS 26.0. Results: Paired samples t-tests showed that the culture change intervention significantly improved the quality of life of the residents. There were significant differences in the scores for privacy at pretest (M=2.63) and posttest (M=3.21); autonomy at pretest (M= 3.20) and posttest (M= 3.43); relationships at pretest (M= 2.88) and posttest (M= 3.36); individuality at pretest (M=3.26) and posttest (M=3.61). An average score for person-centered climate (M=6.25) were significantly higher than that of pretest (M=5.96) (p <.01). Conclusions and Implications: This study suggests that providing support and education to nursing home staff about the culture change strategies is essential to provide personalized services to nursing home residents and encourage residents to participate in the decision-making process.

